# Point prevalence survey on antibiotic use in the hospitals of Mauritius

**DOI:** 10.3389/frabi.2022.1045081

**Published:** 2023-01-09

**Authors:** Lovena Preeyadarshini Veerapa-Mangroo, Harena Rasamoelina-Andriamanivo, Mohammad Iqbal Issack, Eric Cardinale

**Affiliations:** ^1^ Communicable Disease Control Unit, Ministry of Health and Wellness, Port Louis, Mauritius; ^2^ Surveillance Epidemiologiques et Gestion des Alertes (SEGA), One Health network, Ebene, Mauritius; ^3^ Epidemiologic Surveillance and Response Unit, Indian Ocean Commission, Ebene, Mauritius; ^4^ Bacteriology Department, Central Health Laboratory, Victoria Hospital, Candos, Mauritius; ^5^ Research Unit- Animal Health Territories Risks Ecosystems (ASTRE), French Agricultural Research and International Cooperation Organization (CIRAD), Montpellier, France

**Keywords:** point prevalence survey, antibiotic use, hospitals, epidemiology, Mauritius

## Abstract

**Background:**

This study aims at determining the antibiotic prescribing pattern in admitted patients in the regional public hospitals of Mauritius.

**Methods:**

A Point Prevalence Survey (PPS) on antibiotic use according to the World Health Organization Methodology for PPS on antibiotic use in hospitals, was carried out in 3 secondary public hospitals. Data was collected in February 2018 for Hospital 1 and in April-May 2019 for Hospital 2 and Hospital 3. Eligible inpatients were those who were hospitalized in the ward at 8.00 a.m. on the day of the survey.

**Results:**

Among 915 inpatients, 482 (53%) were treated with 753 therapies or prescriptions of antibiotics, averaging 1.6 therapies or prescriptions per patient. Among those treated with antibiotics, an average of 88 patients (55%), 58 patients (36%) and 15 patients (9%) were given 1, 2 and 3 or more antibiotics respectively. The highest proportion of inpatients treated with antibiotics was among those with community acquired infections (n=243, 50%) followed by surgical prophylaxis (n=191, 40%). In the three hospitals, it was observed that third generation cephalosporins (ceftriaxone, cefotaxime), amoxicillin, metronidazole (parenteral) and ciprofloxacin accounted for more than 75% of total prescriptions and sixteen per cent (16%) of patients had an Antibiotic Susceptibility Testing report before prescription of antibiotics.

**Conclusion:**

This study provides valuable information on antibiotic use in the country. Several misuses have been identified such as the excessive use of antibiotics for surgical prophylaxis, the high use of third generation cephalosporins and of the WATCH category of antibiotics. It also demonstrates a low percentage of Antibiotic Susceptibility Testing prior to prescription of antibiotics. This investigation shows that there is now a pressing need to repeat the Point Prevalence Survey on antibiotic use in hospitals in future whilst extending the survey to the private healthcare system to get a complete picture concerning antibiotic use in Mauritius.

## 1 Introduction

Antimicrobial resistance (AMR) has today become a matter of global concern. It has been proclaimed by the World Health Organization (WHO), as one of the top 10 global public health threats (World Health Organisation Antimicrobial Resistance:, 2021). In May 2015, during the Sixty-Eighth World Health Assembly, the global action plan on antimicrobial resistance was adopted (World Health Organisation, 2015a). One of the five objectives of the plan is to optimize the use of antimicrobial medicines (World Health Organisation, 2015b).

One of the main drivers of AMR is the misuse of antimicrobials. Inappropriate use of antibiotics is indeed, accelerating the development of new resistance mechanisms throughout the globe, threatening our ability to treat common infectious diseases and hence, resulting in expensive and prolonged healthcare and sometimes disability or even death (World Health Organisation Antimicrobial Resistance:, 2021). The link between antibiotic resistance and use has been documented in several studies in different countries (Ballow and Schentag, 1992; Melo Cristino, 1999; Kaye et al., 2000; Goossens et al., 2005). Tracking antibiotic use patterns in one’s country is therefore essential to optimize antibiotic prescribing and eventually reduce antibiotic resistance in the country.

The tropical island of Mauritius is a multi-ethnic society with a population of 1.22 million (Ministry of Health and Wellness, 2018a). The Mauritius healthcare system consists of a public and a private sector. The public sector in Mauritius comprises a primary healthcare level which consists of 141 health centers, a secondary healthcare level which consists of five regional hospitals, and a tertiary healthcare level which consists of eight specialized hospitals. The public health sector of Mauritius is completely free of charge.

Concerning the distribution of antibiotics in the public sector, medications are distributed to the different hospitals and health centers of Mauritius and given freely to patients when they present themselves at the government pharmacy with a prescription from the treating doctor. Antibiotic resistance data from the Central Health Laboratory shows that resistance in hospitalized patients of E. coli to cefotaxime had drastically increased from 18% to 46% and resistance to ciprofloxacin from 24% to 58% from 2005 to 2014 (Issack et al., 2007). Another study carried out in the Intensive Care Unit of a hospital shows a higher antibiotic use in patients infected with Multi-Drug Resistance organisms (Nuckchady and Boolaky, 2020). This study therefore aims at determining the antibiotic prescribing pattern in admitted patients in three different hospitals in Mauritius. These valuable information on the use of antibiotics in Mauritius will enable the formulation of appropriate and contextualized protocols and stewardship plans for the country. This in turn will help in the prevention of further resistance of bacteria to antibiotics.

## 2 Materials and methods

A point prevalence survey (PPS) of antibiotic use was carried out in three secondary public hospitals in Mauritius. The WHO methodology for PPS on antibiotic use in hospitals was used for the survey (World health organisation methodology for point prevalence survey on antibiotic use in hospitals, technical document, 2019). A training of trainer’s workshop on PPS survey was initially organized by WHO and the Ministry of Health and Wellness of Mauritius. This was then followed by a 2-day workshop led by the Ministry of Health to train the team at each hospital prior to the data collection. In Hospital 1, the survey was carried out in February 2018 while in Hospital 2 and Hospital 3, the surveys were carried out in April-May 2019. Hospital 1 has a bed capacity of 618 beds and a catchment area population of 314, 796, hospital 2 has a bed capacity of 443 beds and a catchment area population of 180, 482 while hospital 3 has a bed capacity of 495 beds and a catchment area population of 226, 377 (Ministry of Health and Wellness, 2018a; Ministry of Health and Wellness, 2018b). The study in Hospital 1, being a pilot study, 1 in 2 eligible patients were selected for the survey. In the other 2 hospitals, all eligible inpatients were selected for the study. The director of the hospital was notified one week prior to the PPS survey.

Eligible inpatients were those who were hospitalized in the ward at 8.00 a.m. on the day of the survey (excluding those admitted to the ward after 8.00 a.m.). All inpatients meeting the criteria mentioned above were included in the survey whether they were on antibiotics or not. All day care patients were excluded.

Antibiotics were captured from patient notes in the original form and were classified according to the International Non-proprietary Names (INN), the Anatomical Therapeutic Chemical classification (ATC) system and the WHO AWARE category; ACCESS, WATCH and RESERVE groups (World Health Organisation Antibiotics Portal, 2017). Only the systemic antibiotics that the patients were currently taking, were included in the survey.

Topical and ophthalmologic antibiotics were excluded. Data was collected by reviewing the patients’ medical records and data was entered in five different questionnaires namely, ward data, patient data, antibiotic data, antibiotic susceptibility test/microorganisms and indication data. The data was entered on EPI Info questionnaire and Microsoft Excel was used for data analysis. The Ethics Committee of the Ministry of Health and Wellness gave approval for carrying out this study.

## 3 Results

### 3.1 Distribution of inpatients by age and comorbidities

The number of patients included in the survey in the 3 regional hospitals was 915; 276 patients in Hospital 1, 316 in Hospital 2 and 323 in Hospital 3. The average proportion of female patients was 52% while that of admitted male patients was 48%. In all 3 regional hospitals, the proportion of admitted cases per inhabitants was found to be greater in those less than 1 year (9-13 per 10000 population) followed by those above 60 years (5-6 per 10000 population).

Concerning the prevalence of comorbidities in the admitted patients, an average of 38% of the patients had hypertension, 36% had diabetes mellitus, 16% had ischemic heart disease, 9% had other diseases such as renal impairment, bronchial asthma and cancer. The records showed only 1% of the admitted patients were infected with the Human Immunodeficiency Virus (HIV).

### 3.2 Distribution of inpatients by administration route and invasive devices

Regarding the invasive devices used, the overall trend was quite similar among all three hospitals. An average of 84% had a peripheral vascular catheter, 15% had a urinary catheter, 4% had a central vascular catheter while 3% were intubated. The administration route of the antibiotics with the highest proportion (81-89%) in all 3 hospitals was the parenteral route, followed by the oral route (11-19%). Inhalation and rectal route accounted for less than 1%.

### 3.3 Proportion of patients administered with antibiotics:

In all 3 hospitals, among 915 inpatients, 482 (53%) were treated with 753 therapies or prescriptions of antibiotics, averaging 1.6 therapies or prescriptions per patient (**Table 1**). Among those treated with antibiotics, an average of 88 patients (55%), 58 patients (36%) and 15 patients (9%) were given 1, 2 and 3 or more antibiotics respectively.

**Table 1:** Prevalence of antimicrobial use by ward type in hospital 1, 2 and 3.

	Ward Type	Total number of patients, n	Number of patients treated with antibiotics, n	Proportion of patients on antimicrobials (%)	Total number of prescriptions/therapies	Average number of prescription/therapies per patient on antibiotics
**Hospital 1**	Paediatric Medical Ward	18	9	50%	13	1.4
	Adult Medical Ward	103	54	52%	77	1.4
	Adult Surgical Ward	124	72	58%	114	1.6
	Adult High-Dependency Ward	5	3	60%	3	1.0
	Adult ICU	26	13	50%	16	1.2
	All Wards	276	151	55%	223	1.5
**Hospital 2**	Paediatric Medical Ward	27	20	74%	42	2.1
	Adult Medical Ward	163	42	26%	57	1.4
	Adult Surgical Ward	120	80	67%	134	1.7
	Adult ICU	6	4	67%	5	1.3
	All Wards	316	146	46%	238	1.6
**Hospital 3**	Paediatric Medical Ward	16	10	63%	13	1.3
	Adult Medical Ward	204	98	48%	149	1.5
	Adult Surgical Ward	93	68	73%	115	1.7
	Adult ICU	10	9	90%	15	1.7
	All Wards	323	185	57%	292	1.6
**All facilities**	Paediatric Medical Ward	61	39	64%	68	1.7
	Adult Medical Ward	470	194	41%	283	1.5
	Adult Surgical Ward	337	220	65%	363	1.7
	Adult high-dependency and ICU	47	29	62%	39	1.3
	All Wards	915	482	53%	753	1.6

Results also showed 52% of used antimicrobials to be in the WATCH category and 46% in the ACCESS category. RESERVE group accounted for only 2% among the 753 total therapies and prescriptions.

### 3.4 Use by classes of antibiotics

**Figure 1:**
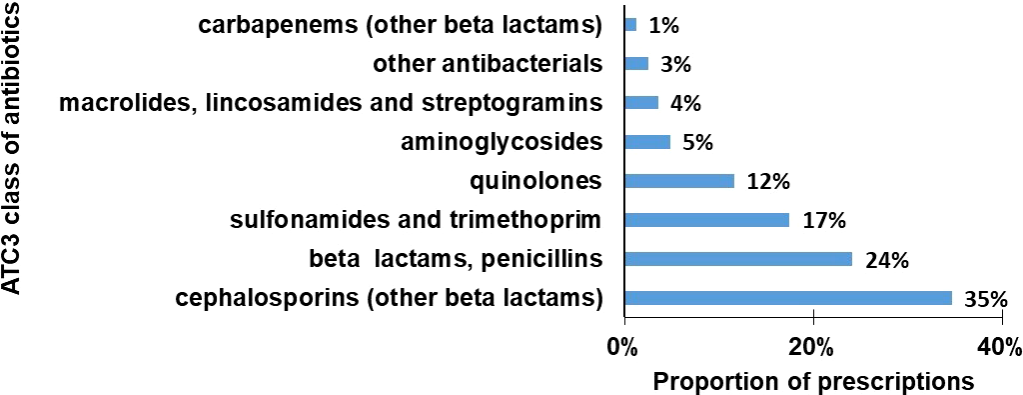
Average proportion of prescriptions done by class of antibiotics, ATC3 (%).

The antibiotics were classified according to Anatomical Therapeutic Chemical 3 (ATC 3), except for other beta lactams which had been subdivided into cephalosporins and carbapenems. The overall trend of choice of antibiotics at ATC 3 level was quite consistent across the facilities. Cephalosporins in the other beta lactams category were the most commonly used antibiotics (35%), followed by beta lactams, penicillins (24%), sulphonamides and trimethoprim (17%) and quinolones (12%) (**Figure 1**).

### 3.5 Indications for antibiotic use

**Figure 2:**
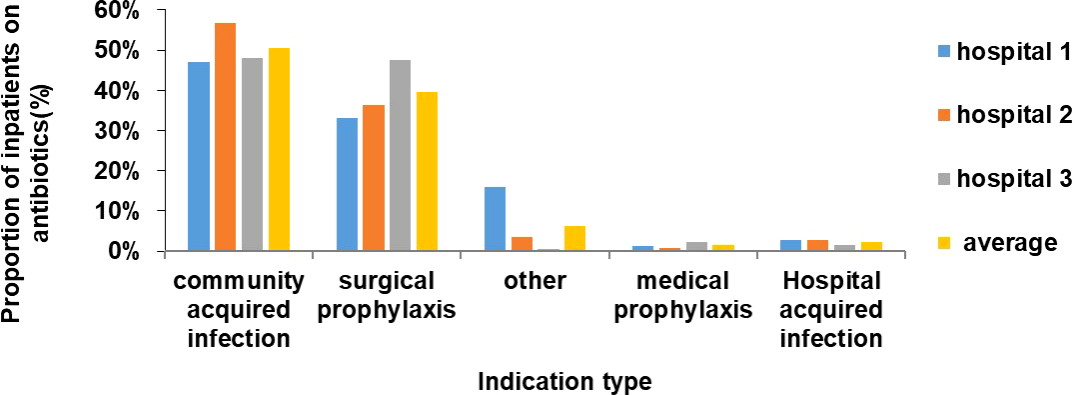
Proportion of inpatients on antibiotics by indication type at the hospitals (%).

**Figure 3:**
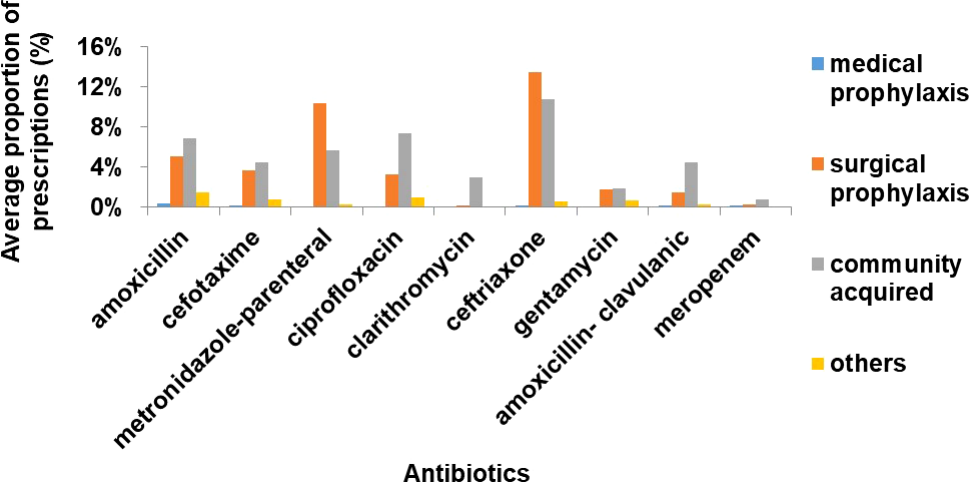
Average proportion of antibiotic prescriptions by indication type (ATC 5 level) (%).

The highest proportion of inpatients treated with antibiotics was those with community acquired infections (n=243, 50%) followed by surgical prophylaxis (n=191, 40%) (**Figure 2**). Ceftriaxone/cefotaxime, amoxicillin, metronidazole (parenteral) and ciprofloxacin were the most commonly used antibiotics in surgical prophylaxis and community acquired infection in all 3 hospitals (**Figure 3**).

The number of doses of antibiotics administered to those categorized as surgical prophylaxis was determined. Above 70% of the inpatients were given multiple doses of antibiotics on more than one day, about 20% were given multiple doses in one day and 5% had only one dose. The same trend was observed in all 3 hospitals.

### 3.6 Most common diagnosis for antibiotic use

**Figure 4:**
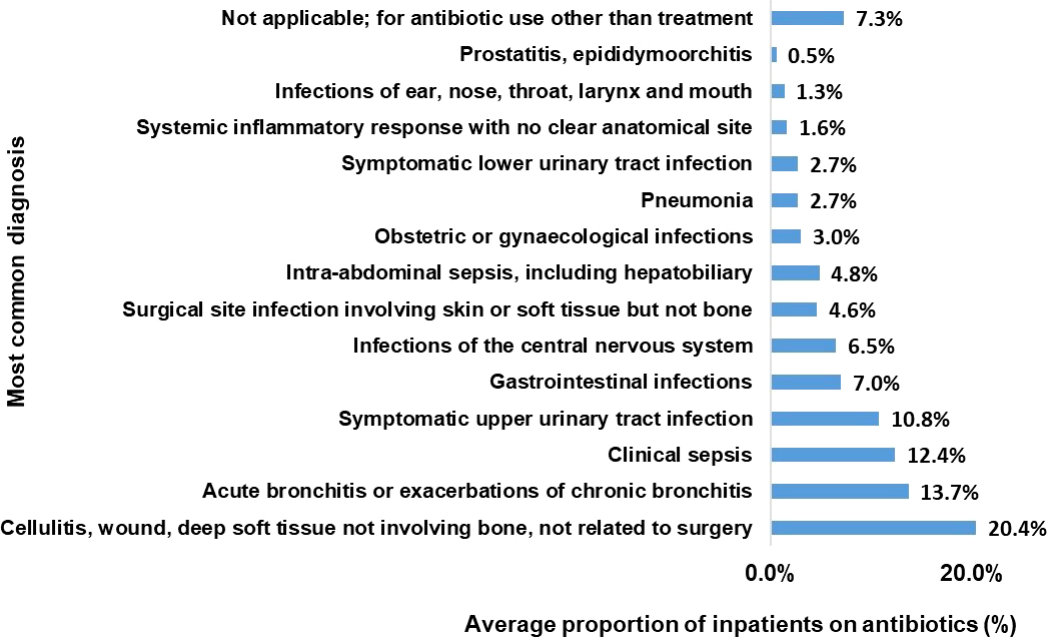
Proportion of the most common diagnosis among the inpatients on antibiotics (%).

On average, the most common diagnosis for antibiotic use was cellulitis, wound, deep soft tissue injury not involving bone and not related to surgery (20%). Acute bronchitis categories came second 14% followed by clinical sepsis and upper Urinary Tract Infection at 12 and 11% respectively. (**Figure 4**).

### 3.7 Most common antibiotics used overall (at the ATC5 level)

**Figure 5:**
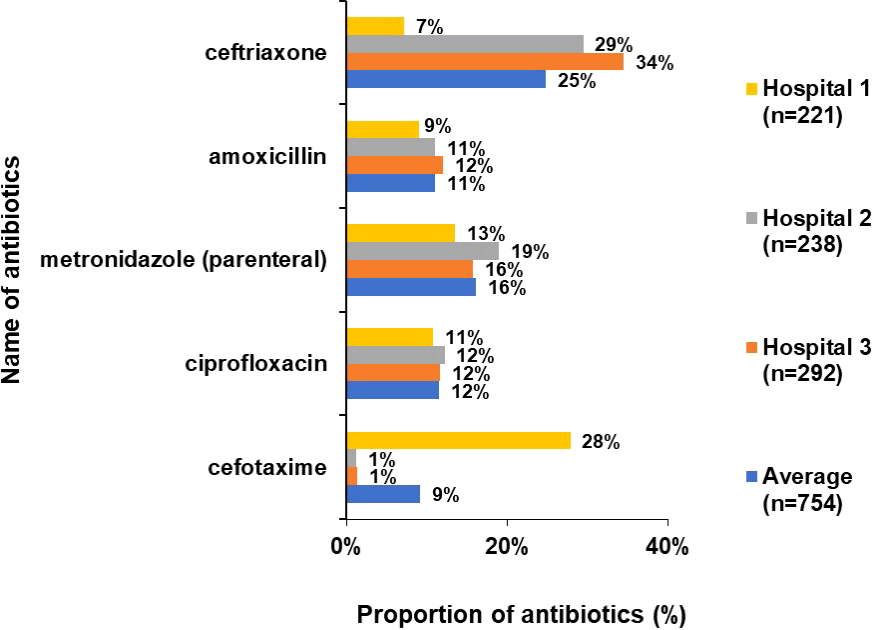
Proportion of commonly used drugs (ATC 5 level) accounting for 75% of total antibiotic use (%).

Overall, third generation cephalosporins (ceftriaxone, cefotaxime), amoxicillin, metronidazole (parenteral) and ciprofloxacin accounted for more than 75% of total prescriptions. While Hospital 2 and 3 had the same trend in the ATC5 level antibiotics, Hospital 1 showed very high cefotaxime use (28%) instead of ceftriaxone (7%). (**Figure 5**).

In Hospital 1,2 and 3, 19 patients (13%, 19/151), 35 patients (24%, 35/146) and 25 patients (13.5%, 25/185) respectively had an Antibiotic Susceptibility Testing (AST) report before prescription of antibiotics. On average, 16% of patients had an AST done before prescription of antibiotics in the 3 hospitals.

## 4 Discussion

The aim of this survey was to determine the antibiotic prescribing pattern in 3 regional hospitals of Mauritius. No similar survey has been done before in the country. The findings show a more or less similar trend in all the 3 hospitals regarding the gender and age of admitted patients, the use of invasive devices, the administration route of antibiotics and the prevalence of comorbidities. Mauritius being a small country, the socioeconomic status, the climate, comorbidities and demographic pattern of the population residing in each region or catchment area of hospitals 1, 2 and 3 are more or less similar. The prevalence of comorbidities calculated in the study were comparable with the prevalence of non-communicable diseases in the country (Ministry of Health and Wellness, 2018a). The population of Mauritius has a high prevalence of diabetes mellitus and hypertension. It is not surprising therefore that the most common diagnosis for the use of antibiotics was in patients with cellulitis (20%). Several studies have shown the high tendency of patients with diabetes mellitus to develop cellulitis (Bristow and Spruce, 2009; Zacay et al., 2021). Patients at age extremes are more vulnerable to diseases and infections and might explain the higher admission in those age groups.

Hospital 3 and 1 had both a greater proportion of admitted patients administered with antibiotics as well as a higher annual number of admissions compared to Hospital 2 (Ministry of Health and Wellness, 2018a). The higher percentage might also be due to the different prescribing habits of the doctors in the 3 hospitals.

In Hospital 1, the adult surgical ward had the highest average number of prescriptions/therapies per patient on antibiotics while in Hospital 2, the pediatric ward had the highest proportion and in Hospital 3, the highest proportion was in adult ICU and adult surgical ward. The pediatric ward of Hospital 2 was a mixed ward where both surgical patients and medical patients were admitted. A higher number of prescriptions in surgical pediatric patients than in medical children may explain the high proportion in Hospital 2. Hence, on average, the proportion of antibiotic prescriptions was higher in the surgical wards. Furthermore, proportion of patients on antibiotic due to surgical prophylaxis was found to be high (33-48%) and more than 60% of them were given multiple doses of antibiotics on more than one day. A similar high use of antibiotics in surgical prophylaxis has also been observed in Europe, Saudi Arabia, Turkey, Ghana, Egypt, India and China (Usluer et al., 2005; European Centre for Disease Prevention and Control, 2011-2012; Talaat et al., 2014; Xie et al., 2015; Matar et al., 2016; Labi et al., 2018; Singh et al., 2019). The WHO and several studies recommend the use of one dose of antibiotic just before the surgical procedure with appropriate choice of antibiotic and clean aseptic conditions during the operation (Nichols, 2004; Hawn et al., 2013; World Health Organisation, 2018). The reason for this excessive use of antibiotics after surgery in Mauritius might be the high rate of Surgical Site Infections (SSI) and poor Infection Prevention Control (IPC) measures. In this study, the proportion of SSI was found to be 5% while in 1993 and 2021, two studies done in a regional hospital of Mauritius found a higher incidence of 8.2% and 17% respectively (Jepsen et al., 1993; Nuckchady, 2021). A follow up of patients after surgery is important to get more accurate data and might explain the low proportion of SSI obtained in this point prevalence study. The high SSI incidence might explain the behavior of the doctors in using several doses of antibiotics for surgical prophylaxis after surgery. A surveillance of SSI and IPC is therefore important to measure the burden in Mauritius. This will help in formulating and implementing National IPC guidelines and antibiotic stewardship protocols in all hospitals.

The proportion of patients on antibiotics due to medical prophylaxis on the other hand was found to be surprisingly very low in this study (<2%) compared to Europe (11%), Egypt (23%) and India (17%) (European Centre for Disease Prevention and Control, 2011-2012; Talaat et al., 2014; Singh et al., 2019). Given the high rates of admitted patients with non-communicable diseases as diabetes, hypertension, ischemic heart disease in these cohorts as well as high incidence of nosocomial infections in one hospital of Mauritius, it is surprising that antibiotics given for medical prophylaxis is low (Nuckchady, 2021). A reason explaining this discrepancy in data might be due to the fact that in Europe and India, data was collected in acute care settings and tertiary hospitals respectively while data was collected in secondary hospitals in Mauritius. Another reason for this difference of data with other countries might be due to improper documentation in patients’ files as the reason for administration of antibiotics is often not specified.

A PPS on antimicrobial use in Europe, China, Turkey (Matar et al., 2016; Labi et al., 2018; Singh et al., 2019) showed adult ICU to have higher antibiotic therapies than Mauritius. Here also, the higher rate of antibiotic therapies might be due to the different hospital settings of Europe and Turkey where the PPS surveys were carried out in acute care hospitals and tertiary hospitals respectively. The data should therefore be compared with caution. In Mauritius, this greater rate was observed in Hospital 3 only. The lower use of antibiotics in the ICUs of Hospital 1 and 2 may be due to bacterial culture and susceptibility testing being done regularly for every patient in the ICUs. Hence, only those antibiotics which were sensitive to the pathogen isolated were administered to the patients.

The use of meropenem was very low at less than 2% in the hospitals. An antibiotic susceptibility test is usually recommended by the hospital pharmacists before dispensing meropenem and might explain its low proportion used in the study.

A high proportion (84%) of patients with peripheral vascular catheter (PVC) was seen in the 3 hospitals. The data is comparable to the PPS on antibiotic use done in several countries of Europe, Saudi Arabia, Ghana, Democratic Republic of Congo, Botswana and China (European Centre for Disease Prevention and Control, 2011-2012; Matar et al., 2016; Wambale et al., 2016; Labi et al., 2018; Paramadhas et al., 2019; Singh et al., 2019) Though it can be argued that PVC helps in providing urgent care in case of deterioration of the patient, clear guidelines should be elaborated on the use of PVC along with the administration of oral v/s parenteral treatment. The proportion of antibiotics given *via* parenteral route was 85%. This data is comparable to proportion reported in Europe (71.9%), Saudi Arabia (80.6%) and China (98%) and (European Centre for Disease Prevention and Control, 2011-2012; Xie et al., 2015; Matar et al., 2016). The increased use of catheter and unnecessary use of vascular access can lead to the development of HAI and as a result, oral antibiotics should be preferred if patient is stable and tolerating orally. More information on these variables is needed to assess the patients in the study.

The proportion of inpatients with HAI indication was found to be surprisingly very low at 2-3% in these cohorts. Two studies done in 1993 and 2021 showed the prevalence of HAI to have increased from 4.9% to 18% in one hospital of Mauritius (Jepsen et al., 1993; Nuckchady, 2021). However, the results have to be compared with caution as the collection of data may not have been uniform in the different studies. Mauritius is seen to have a lower proportion of HAI compared to Europe (6%), India and several countries of Africa (European Centre for Disease Prevention and Control, 2011-2012; Talaat et al., 2014; Labi et al., 2018; Paramadhas et al., 2019; Singh et al., 2019). It is important to keep in mind that the survey in Europe, India and some countries of the African region were carried out in different healthcare settings as acute care and tertiary hospitals which might explain this difference in data. Moreover, this lower HAI proportion in Mauritius could also be due to underestimation and lack of documentation in the patient files. The PPS survey only collects data at one point in time so that there is no follow up of patients from admission to discharge. HAI in the inpatients could be happening at a later stage and therefore be missed. Proper reporting of HAI in the files and follow up of the patient till discharge will help in getting accurate data on HAI and in assessing IPC measures in the hospitals.

As for the AWARE categorization (World Health Organisation Antibiotics Portal, 2017), around half of total use were in the ACCESS or WATCH category while only 1-4% was in the RESERVE group. Since the data was collected from inpatient records at regional hospitals where more severe conditions are usually treated, the proportion of ACCESS and WATCH antibiotics seems acceptable. However, since the country has not elaborated its own therapeutic guidelines of antibiotic use, their adoption in both public and private facilities could optimize the use of antibiotics.

A third-generation cephalosporin (ceftriaxone or cefotaxime), amoxicillin, metronidazole and ciprofloxacin accounted for 75% of the total antibiotic prescriptions. This data is comparable to PPS studies done in Europe, Africa and China (European Centre for Disease Prevention and Control, 2011-2012; Xie et al., 2015; Wambale et al., 2016; Labi et al., 2018; Paramadhas et al., 2019). Cefotaxime was most commonly used in Hospital 1 while ceftriaxone was highest in Hospital 2 and 3. The doctor prescribing habits or the temporary non-availability of ceftriaxone at one hospital might be an explanation for the choice of antibiotic in the hospitals.

It is important to note that an AST was done for only a small percentage (16%) of inpatients prior to prescription of antibiotics. Appropriate guidelines should be elaborated so that although antibiotics can be started in critically ill patients, an AST should be sent prior to the administration of antibiotics belonging to the WATCH and RESERVE category.

Though the study provides important data, it is important to keep in mind the limitations of the study when interpreting the results. The data collection in Hospital 1 was carried out in February 2018 while the data collection in Hospital 2 and 3 was carried out in April-May 2019. The difference in season, month and year of data collection might explain differences in the data. According to the Mauritius Meteorological Services, February is identified as the warmest and wettest months of the year while May is known as the transition months (Mauritius Meteorological Services). Furthermore, categorization of the different wards was not always accurate. When the wards were full, a patient from one ward were transferred to another ward category so that the wards are often mixed wards. Also, according to the protocol, only antibiotics that the patient was currently taking on the day of the data collection, were included, underestimating the antibiotic prescriptions obtained by the patient in all. Although this PPS survey which is a standard protocol, is crucial for comparison of results with time and across countries, it is important to highlight that following a cohort of patients from admission till discharge in the hospitals would have provided more accurate data on HAI, SSI and more details on duration and different classes of antibiotics administered to the patient. This can be incorporated in the future PPS surveys on antibiotic use so that not only results can be compared to see the progress but also, a cohort of patients in the surgical wards and medical wards can be followed till discharge or death.

Also, the healthcare staff at the hospitals were aware of the survey on antibiotic use prior to the data collection and this might have influenced the documentation in the patients’ files and led the doctors to prescribe antibiotics more carefully. Regular surveys on antibiotic use without prior notification to the healthcare staff will enable us to have more accurate data.

This study provides valuable information on antibiotic prescribing pattern in the country which will help in formulating contextualized antibiotic stewardship program. Afterwards, it would be imperative to continue the PPS survey on antibiotic use in at least one regional hospital each year for monitoring and evaluation of the implementation of the stewardship protocols and to disseminate the results in the hospitals to improve awareness of the healthcare workers. The study can be used in the future with the results of culture and antibiogram of the samples obtained in these hospitals for antibiotic stewardship program. The surveys on antibiotic use could then be extended to the private healthcare system of the country to get a complete picture concerning antibiotic use. Addressing the issues on antibiotic use is essential for each country to be able to fight antibiotic resistance.

## 5 Conclusion

The PPS on antibiotic use provides important information that can be used to formulate an appropriate antibiotic stewardship program in Mauritius as well as a standard way of collecting data so that it can be compared across countries. No similar survey on antibiotic use has been done before in Mauritius. Several misuses of antibiotics have been identified in the 3 regional hospitals such as the excessive use of antibiotics for surgical prophylaxis, the high use of third generation cephalosporins and of the WATCH category of antibiotics. The survey also demonstrates an extremely low percentage of AST done prior to prescription of antibiotics. The survey should be continued each year after the implementation of an antibiotic stewardship program to constantly review its effectiveness so that we are able to tackle antibiotic resistance in Mauritius.

## Data availability statement

The raw data supporting the conclusions of this article will be made available by the authors, without undue reservation.

## Author contributions

LV-M collected, analyzed, and interpreted the data. LV-M conceived the original paper and provided a rough draft. EC, MI and HR-A interpreted the data, checked the paper for consistency, corrected the language and checked the references for consistency and accuracy. All authors contributed to the article and approved the submitted version.
